# Analysis and Economic Evaluation of the Use of Recycled Polyamide Powder in Masonry Mortars

**DOI:** 10.3390/polym12112657

**Published:** 2020-11-11

**Authors:** Miguel A. Salas, Heriberto Pérez-Acebo, Verónica Calderón, Hernán Gonzalo-Orden

**Affiliations:** 1 Department of Architectural Construction and Construction and Land Engineering, University of Burgos, 09001 Burgos, Spain; masalas@ubu.es (M.A.S.); vcalderon@ubu.es (V.C.); 2 Mechanical Engineering Department, University of the Basque Country UPV/EHU, 48013 Bilbao, Spain; 3 Department of Civil Engineering, University of Burgos, 09001 Burgos, Spain; hgonzalo@ubu.es

**Keywords:** polyamide, waste material, waste polyamide, mortar, lightweight mortar, compressive strength, sustainable material

## Abstract

Due to the considerable amount of waste plastics and polymers that are produced annually, the introduction of these waste products in construction materials is becoming a recurrent solution to recycle them. Among polymers, polyamide represents an important proportion of polymer waste. In this study, sustainable and lightweight mortars were designed and elaborated, substituting the aggregates by polyamide powder waste. Mortars were produced with various dosages of cement/aggregates, and the polyamide substitutions were 25, 50, 75, and 100% of the aggregates. The aim of this paper is to determine the density and the compressive strength of the manufactured mortars to observe the feasibility for being employed as masonry or rendering and plastering mortars. Results showed that with increasing polymer substitution, lower densities were achieved, ranging from 1850 to 790 kg/m^3^ in modified mortars. Mortars with densities below 1300 kg/m^3^ are cataloged as lightweight mortars. Furthermore, compressive strength also decreased with more polyamide substitution. Obtained values in recycled mortars were between 15.77 and 2.10 MPa, but the majority of the values (eight out of 12) were over 5 MPa. Additionally, an economic evaluation was performed, and it was observed that the use of waste polyamide implies an important cost reduction, apart from the advantage of not having to manage this waste material. Consequently, not only the mechanical properties of the new recycled materials were verified as well as its economic viability.

## 1. Introduction

At present, there is an international agreement about the necessity of sustainable development with the aim of a more efficient employment and management of the limited natural resources, which includes the promotion of recycling and reuse of waste materials [1]. For plastics and polymers, which are two of the main industrial byproducts and home waste materials [2], various processes are being conducted to reuse and recycle them, such as mechanical recycling (secondary polymers are obtained through mechanical processes), chemical recycling (monomers are recovered to be employed as new virgin polymers or are transformed in other useful materials), and energy recovery (energy is obtained from the combustion of post-consumer plastics) [3,4,5,6,7]. Additionally, the introduction as fillers in other materials is becoming a possible solution for plastic and polymeric waste materials, especially in construction materials, with examples of reuse in various structural materials, such as concrete [8,9,10,11,12,13], mortars [14,15,16,17,18], bituminous materials for pavements [19,20,21,22,23,24,25,26,27,28,29,30], and gypsum [31,32,33,34,35,36].

Polyamides are a widely employed thermoplastic polymer. Their main applications are fibers for the textile industry, ropes, toothbrushes, technical parts of vehicles, and gears [5,37], taking advantage of their main properties: thermal stability, chemical resistance, low viscosity before curing, low yield stress, and stress relief. For example, one of the main applications is laser sintering, where polyamides are used as raw material for manufacturing functional parts due to their low density and good mechanical properties. However, after a limited number of times, polyamides cannot be further used, since their properties are not maintained. Of the 61.8 million tons of plastics produced in Europe in 2018, around one million tons was polyamides [38]. Although there are not specific data about the polyamide waste in the laser sintering industry, it can be estimated that the waste amounts to 20% of the total, i.e., representing 200,000 tons of waste per year worldwide [39].

Consequently, there is a need to find a solution for the valuation and employment of this waste product. Efforts are being made to reuse or recycle waste polyamide [40,41,42]. With regard to the use of polyamide powder in construction materials, some previous analyses have been conducted about the characterization of recycled mortars with polyamide powder waste, analyzing their durability and their microstructure [39,43,44]. These previous papers showed that introducing polyamide powder waste is suitable for mortars by analyzing the properties of fresh and hardened mortar like workable life, water retention, water vapor permeability, porosity, adhesion, thermogravimetry, and durability (by determining the frost resistance, the resistance to ageing by thermal shock and to salt crystallization, and by testing the potential expansion of aggregates from hydration reactions). Additionally, the microstructure was analyzed by mercury intrusion porosimetry and by scanning electronic microscopy, and the macrostructure using computerized axial tomography.

Therefore, once that the possible employment of polyamide powder waste in masonry mortars has been preliminary verified for aggregate replacement, it is necessary to go one step further and check if produced new mortars fulfill the requirements of compressive strength that masonry mortars must verify. Thus, the applicability of this sustainable material would be verified and justified for real masonry works.

Hence, the objective of this paper is to know the variation of the density and the compressive strength of the mortars with recycled polyamide with varying dosage of cement and aggregates and increasing percentage of substitution of aggregates by polyamide powder waste. Additionally, an economical evaluation was conducted to verify if the employment of this waste material offers a production cost reduction.

## 2. Materials and Methods

### 2.1. Employed Materials

Employed materials in this research were aggregates, cement, water, and waste polyamide. Aggregates came from a quarry, which can be classified as sand 0/4, i.e., sieved between 0 and 4, and it was characterized according to EN-13139 standard [45]. Table 1 shows the physical characteristics of the aggregates, obtained from laboratory tests.

Employed water came from the municipal water system of the Council of Burgos (Spain), and its analysis is exposed in Table 2.

The cement used in this work was a CEM IV/B (V) 32.5 N, which is a puzzolanic cement type IV, with the addition of siliceous flying ash, low content of clinker, a normal uniaxial compressive strength (UCS) of 32.5 MPa, and an ordinary initial mechanical strength. It has a density of 3030 kg/m^3^, and its chemical composition is 45–64% clinker (K), 36–55% fly ash (V), and 0–5% minority constituents, according to Standard EN 197-1:1994 [49]. It is common cement in masonry works and in roads for soil stabilization and soil cement [50,51]. Table 3 shows the properties of the cement.

The polyamide powder waste is obtained from the waste raw material produced in an industrial process of laser sintering. Its gradation is below 1 mm, and its real density is 1070 kg/m^3^. The characteristics of the polyamide PA 2200, according to the producer’s file, can be observed in Table 4.

### 2.2. Preparation of Mortar Samples

Traditional reference mortars, which are usually employed in masonry works, are produced following the proportions that are imposed in projects and works, which are based on experience, without standards to define them.

Reference mortars, i.e., mortars without polyamide, are produced according to the cement/aggregates (C/A) proportions in the volume included in Table 5: 1/3, 1/4, and 1/6.

The ratio between cement and water determines the workability of the mortar and the final characteristics [61]. The ratio water/cement was fixed to achieve an adequate consistency for masonry works, which is said to be the plastic, with a value of 175 ± 10 mm following the EN 1015-3 standard [62].

The aggregates in the mortars were substituted by an increasing percentage of waste polyamide: 25, 50, 75, and 100% of the aggregates were replaced by polyamide powder waste. Therefore, 5 mortar specimens (with commented substitution of aggregates by polyamides and a reference mortar without substitution) were produced for each of the 3 proportions (1/3, 1/4, 1/6), and, hence, 15 different specimens were produced.

As established in the standard EN 1015-2 [63], the fresh mortar employed in the tests must have the adequate consistency for its use in real works. For masonry works, the mortars that assure an adequate consistency have plastic consistency, denominated as “P”, as defined in Table 6.

The mortar was prepared from dry raw materials, Figure S1a, and water. A mechanic mixing of 5 l of capacity was employed for mixing the mortar, shown in Figure S1b. It was prepared following the procedure established in the standard EN-196-1 [64].

### 2.3. Tests

The densities of the manufactured mortars were calculated and the compressive strength of the specimens was tested.

The density was calculated with the hardened material following the standard EN 1015-10 [65]. The compaction equipment is shown in Figure S2a, and the mold being filled and compacted can be observed in Figure S2b.

All the samples have followed the same procedure. The molds with the fresh mortar were introduced in a curing room, with a constant temperature of 20 °C ± 2 °C and a relative humidity of 95% (Figure S3). After 24 h, they were taken from the molds, and after being conveniently referred, they were introduced again in the curing room to continue the curing process in the same conditions. Specimens have the following dimensions: 40 mm × 40 mm × 160 mm. Twenty-eight days after they were produced, samples were tested.

The compressive strength of the specimens was calculated following the standard EN 998-1 [66] and the standard EN 998-2:2016 [67]. The equipment used for the compressive tests was a universal test machine of the firm Suzpecar, model MEM-101/SDC, shown in Figure S4a. The test was carried out in each of the two parts obtained after a flexural test. The specimen is placed between two plates of 40 mm × 40 mm, shown in Figure S4b. The machine applies a load at a constant speed until the sample is broken.

## 3. Results and Discussion

Table 7 shows all the dosages of the mortars that were manufactured.

It was observed that the quantity of water is higher as the quantity of substituted aggregates increases due to the diameter of the particle of polyamide powder, which is smaller than the aggregates that are substituted. Hence, there is a higher specific surface, which requires a higher quantity of water as the amount of polyamide increases.

### 3.1. Density of the Mortars

The density of the mortars is mainly dependent on the density, gradation, and volume of their components. Moreover, the proportion of water/cement also has an impact on the density, becoming more porous as the proportion increases. The values of density after 28 days are shown in Table 8.

It can be observed that the density of the hardened materials is inversely proportional to the added quantity of polyamide. With a higher percentage of polymer, the requirement of water is higher, which increases the porosity due to the evaporation of water, which justifies the decrease in the density. When the aggregates are totally substituted, the densities decrease 46, 53, and 61% in series I, II and III, respectively.

**Figure 1:**
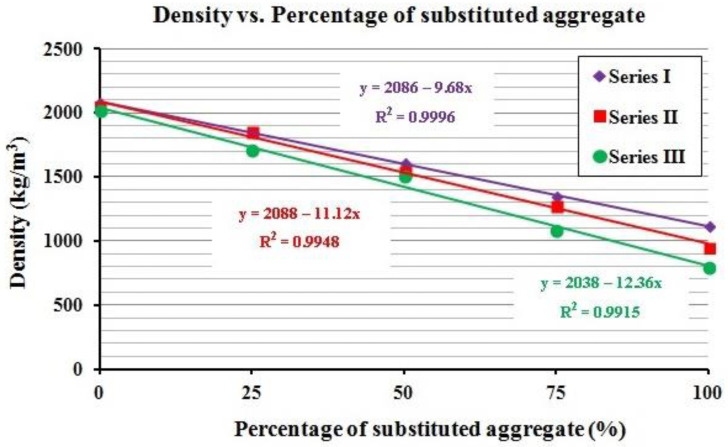
Density vs. percentage of substituted aggregate.

Additionally, the scatter plot of the density vs. the percentage of substituted aggregate was created for each series to analyze the curves that best correlate both variables for each series (Figure 1). A linear relationship was established for all the series, with determination coefficients over 0.99, implying that more than 99% of the variability of the variable can be explained by the models [68,69,70,71]. Moreover, the coefficients of the independent variable (the percentage of substituted aggregates) showed that for each 10% of aggregate replacement, the density decreases between 96.8 to 123.6 kg/m^3^, depending on the mortar series.

From all the tested specimens, there are five with a density below 1300 kg/m^3^. These mortars can be considered as “Light rendering and plastering mortar” according to standard EN 998-1:2016 [66] and “light masonry mortar” according to standard EN 998-2:2016 [67], which could be employed in applications where loads must be reduced.

### 3.2. Compressive Strength

The compressive strength is one of the main parameters to select the type of mortar in works. The compressive strength gives an idea of the internal cohesion of the mortar, indicating its ability to support loads without disaggregation. It is a measure of the mechanical quality of the mortar, and it can be related to other properties such as the adherence or the durability.

The standard EN 998-2:2016 [67] classifies the masonry mortar as a function of its compressive strength at 28 days, as shown in Table 9.

The standard EN 998-1 [65] classifies the rendering and plastering mortars as a function of its compressive strength at 28 days, as shown in Table 10.

For each mortar denomination, six samples were tested. The final value was obtained as the arithmetical mean of the individual values, shown in Table 11. The values of the six specimens that were tested for each denomination are shown in Table S1 in Supplementary Materials. As seen, the variability within the same denomination is low.

The progressive decrease in the compressive strength as the polymer quantity increases is due to the lower density of the polyamide and the increase in water in the mixes with higher proportions of polymer, which leads to a higher porosity in the hardened material and, hence, also to lower strengths. A total replacement of aggregates decreases the compressive strength up to between 61 and 67%, depending on the series.

**Figure 2:**
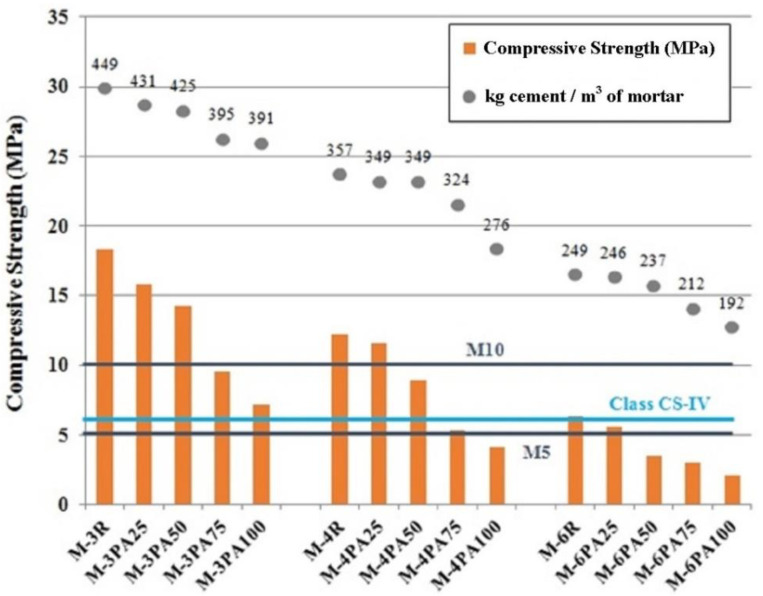
Comparison between the compressive strength values (MPa) and kg cement/m^3^ of mortars.

In Figure 2, it can be observed the compressive strength at 28 days of the mortars with the employed quantity of cement in each denomination.

As seen, although the progressive decrease in the density means an increase in porosity, which results in a reduction in the compressive strength, most of the mortars have a compressive strength over 5 MPa, which is enough for most of the applications of mortars. In Figure 2, three lines were introduced, with the values of 5 and 10 MPa for masonry mortars (M5 and M10) and with the value of 6 MPa for rendering and plastering mortars (Class CS-IV). As indicated in Table 9, the minimum strength for masonry mortars is 1 MPa, a value that is reached by all the tested specimens.

**Figure 3:**
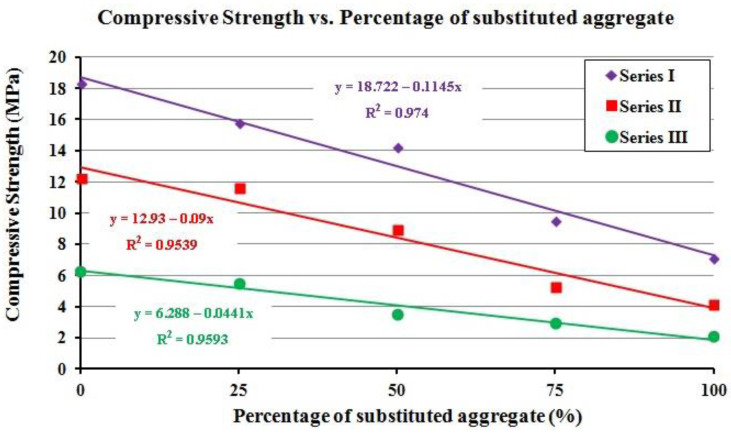
Density vs. percentage of substituted aggregate.

Additionally, the correlation between the compressive strength and the percentage of substituted aggregate was analyzed. The scatter plot of the compressive strength vs. percentage of substituted aggregates showed again that the relationships were linear for the three series, with determination coefficients over 0.95 (Figure 3). Moreover, with a replacement of 10% of aggregates, the compressive strength decrease 1.1 MPa and 0.9 MPa in series I and II, respectively, while in series III, 0.04 MPa.

## 4. Economic Viability

The following factors were considered for the economic viability of mortars with recycled polyamide waste:

- The cost of the raw material until the moment that consumers can buy it.
- The cost of mixing the materials. These costs include the transport to the construction site.
- The cost of placing the material in the construction site.

Therefore, the cost that is similar in traditional mortars and in recycled mortars is not considered, because it does not give additional information about the viability of the proposed models.

### 4.1. Quantitative Viability

The costs of each of the raw materials were obtained as follows:

- The prices of the cement and the aggregates (sand) were asked to various construction material suppliers.
- The price of the water was obtained from the Water Service of the Council of Burgos.
- The price of the polyamide is considered as zero, and it is supposed that it is powder, hence, there is no need for further treatment.

In Table 12, the prices of each of the raw materials are shown, in EUR per kg.

Considering the proportions and required quantities for each of the mortars that were tested, the cost of a m^3^ is determined for each component and for the mortar (Table 13).

Therefore, it can be seen that with a higher proportion of polymer in the mortar, the cost of the final product is reduced due to the simple substitution of the aggregates (with a determined cost) by waste polyamide, which, ideally, is obtained at zero cost, because it does not need any treatment before the addition to the mixture. However, the cost of the transport of the waste polyamide from the place where it is generated to the mortar production plant must be considered as a key factor. This cost must be included in recycled mortars. Obviously, higher distances imply higher costs.

After asking various transport companies, the average cost of the transport of the polyamide would be EUR 0.04 per ton and kilometer. Similarly, using data from various transport companies, another transport cost can be calculated:

- Truck used for the transport: truck with a load capacity of 25 t, with a cost of 40 EUR/h, which includes the vehicle costs (assurance, maintenance costs, etc.), petrol costs, and driver.
- A time of 4 h is estimated for 100 km of outgoing and 100 km of return travel, including uploading and downloading.

For calculating the transport cost, Equation (1) was employed:

*Load* 2 *Distance Price* = *Travel time Hour cost* (1)

where *Load* is the load that the truck can transport (t), *Distance* is the distance of one way of transport (km), *Price* is the price that cost the transport (EUR/t), *Travel time* is the estimated time for outgoing and return travel (h), and *Hour Cost* is the cost per hour of the truck (EUR/h).

Substituting the values in Equation (1),

25 t 2 100 km *Price* = 4 h 40 EUR/h (2)

*Price* (cost) of 0.032 EUR/t/km is obtained, lower than the firstly indicated average price. Taking the higher one, i.e., 0.04 EUR/t/km, the cost of mortars produced in the place of the waste polymer (last column of Table 13) and taking into account the transport of that waste polymer to various distances (Table 14).

As seen, the proposed mortars with waste polyamide have a similar cost, even if long distances are considered. The cost reduction in recycled mortars is similar to the cost of production of those recycled mortars in place of the waste materials, i.e., the transport cost increases slightly the total cost, being almost independent of the distance.

### 4.2. Qualitative Viability

As it can be deduced from the results of the quantitative evaluation, the proposed mortars with waste polyamide are economically feasible by themselves. Nevertheless, the research was completed with other variables, which, although they cannot be measured in net values, have an impact on the added values of these products and must be taken into account.

Initially, it was considered that the waste materials were sent to landfills at zero cost, but, in reality, the treatment of these by-products have a transport and management cost. Hence, this profit must be included to the previous calculations.

Various waste management companies were consulted and the average cost of managing this polymer would be around 60 EUR/t of polyamide. If it is applied to each of the proposed percentages, an additional profit is obtained that must be deducted from the final cost (Table 15).

When the 100% of the aggregates are replaced by polyamide, the mortar becomes very economic, reaching the case that the M-6PA100 mortar has a final cost of practically null.

### 4.3. Mortar Selection

When the cost reduction factors are considered, instead of the mechanical properties of the mixes, it can be deduced that some proportions provide enough compressive strength for being employed as masonry or rendering and plastering mortars, a lower density that contributes to lighten the final load of the building structure, and lower final cost. Table 16 summarizes all the relevant data of the tested mortar specimens: properties of the material and prices of the mortars, according to the distance between where the polyamide powder waste is generated and the mortar production plant and considering the cost of the waste material management (60 EUR/t of polyamide).

The values of Table 16 do not show a unique better solution for mortar selection. It is clear that higher percentages of aggregate substitution lead to lower prices, and mortars with 75 or 100% of replacement would be preferred. However, those mortars would not be useful for some applications. Furthermore, the obtained prices are approximate and can vary from one country to another depending on the cost of the transport and the cost of the waste management. Moreover, masonry mortars are employed in various applications and the requested strength values can vary. Therefore, it is not adequate to identify a unique mortar as the most appropriate one. However, it is worth mentioning the mortar M-3PA75, which is very interesting, because it has a cost of 38.64 EUR/m^3^ (it has a cost reduction in more than 56% when compared to the reference mortar M-3R). It practically reaches a compressive strength of 10 MPa, which is enough for required applications of this type of materials, and a density of 1350 kg/m^3^.

## 5. Conclusions

Apart from the implicit environmental profit that represents the employment of large amounts of waste polymers and the reduction in the employment of non-removable natural resources, this research develops the production of lightweight mortars with waste powder polyamide with additional advantages.

Firstly, the aggregates of the mortars can be substituted partially or completely by polyamide powder waste, producing workable materials as far as the quantity of water that provides an adequate consistency (plastic) is added. Moreover, the density of the specimens decreases considerably as the introduced quantity of waste materials increases, reaching densities of 800 kg/m^3^ in a hardened state, lower than 1300 kg/m^3^, which is considered the maximum value for a lightweight mortar.

Although the compressive strength decreases as more quantity of polymer is introduced, there are some dosages that fulfill the standards in law regarding the mechanical properties when they are employed as masonry or rendering and plastering mortars.

From the economic study the following conclusions can be extracted:

- The final price of the mortar depends on the quantity of each element separately, being the percentage of cement the determinant factor.
- Higher quantities of polyamide imply higher price reduction.
- If the distance from the location of the waste polymer to the point of production is up to 200 km, the price of the mortar is increased by EUR 8, but the saving of the waste management can be up to EUR 66.

In case of recommending a masonry mortar with enough mechanical properties and a competitive price, the mortar M-3PA75 would be selected because its compressive strength is near 10 MPa, and the price reduction, when compared with the reference mortar, is about 35%.

Finally, after verifying the feasibility of the employment of these recycled mortars, in a next step of the research, performance in real works can be checked.

## Figures and Tables

**Table 1:** Physical characteristics of the aggregates.

Test	Standard	Result
Fine content	EN-933-1 [46]	0.17%
Particle size	EN-13139 [45]	0/4
Quality of fine	EN-13139 [45]	Contrasted
Aggregate shape	EN-13139 [45]	Not relevant
Bulk density	EN-1097-3 [47]	1380 kg/m^3^
Real density of the aggregate	EN-1097-6 [48]	2600 kg/m^3^
Porosity	EN-1097-3 [47]	46.92%

**Table 2:** Physical and chemical parameters of the water.

Physical and Chemical Parameters	Units	Values	Physic and Chemical Parameters	Units	Values
Smell	Dilution index	0.0	Sodium	mg/l	2.2
Taste	Dilution index	0.0	Chlorides	mg/l	3.9
Color	mg/l Pt/Co	<1.0	Sulfates	mg/l	5.5
pH	pH units	8.0	Calcium	mg/l	18.0
Turbidity	UNF	0.	Total hardness	°fH	5.0
Conductivity	μS/cm	111.8	Bicarbonate	mg/l	57.6
Nitrates	mg/l	0.7	Iron	mg/l	0.02
Nitrites	mg/l	<0.05	Aluminum	mg/l	0.03
Ammonium	mg/l	<0.01	Copper	mg/l	<0.005
Residual free chlorine	mg/l	0.5	Sum of trihalomethanes	mg/l	<0.02

**Table 3:** Cement properties.

Main Standardized Component	Value	Cement Standardized Specifications	Value
Clinker (K)	45–64%	Sulfate	≤3.5%
Silica fumes (D) ^1^	-	Initial setting time	≥75 min
Natural pozzolana (P) ^1^	-	Final setting time	≤720 min
Calcined natural pozzolans (Q)^1^	-	Expansion	≤10 mm
Siliceous fly ash (V) ^1^	36–55%	UCS at 7 days	≥16 MPa
Calcareous fly ash (W) ^1^	-	UCS at 28 days	32.5 ≤ R ≤ 52.5 MPa
Minority components	0–5%	Puzzolanicity	8 to 15 days
Chlorides	≤0.10%	-	-

^1^ The sum of (D), (P), (Q), (V), and (W) for cements CEM IV must be 36–55%.

**Table 4:** Characteristics of the PA 2200 polyamide according to the supplier’s technical file.

Characteristics	Standard	Value	Units
Average granulometry	Laser curvature	60	μm
Bulk density	DIN 53466 [52]	0.435–0.445	g/cm^3^
Laser sintered density	EOS method	0.9–0.95	g/cm^3^
E tensile modulus	EN ISO 527 [53]	1700 ± 150	MPa
Tensile strength	EN ISO 527 [53]	45 ± 3	MPa
Elongation at fracture	EN ISO 527 [53]	20 ± 5	%
E flexural modulus	ES ISO 178 [54]	1240 ± 130	MPa
Charpy impact test strength	EN ISO 179 [55]	53 ± 3.8	kJ/m^2^
Charpy resilience	EN ISO 179 [55]	4.8 ± 0.3	kJ/m^2^
Izod impact test strength	EN ISO 180 [56]	32.8 ± 3.4	kJ/m^2^
Izod resilience	EN ISO 180 [56]	4.4 ± 0.4	kJ/m^2^
Ball indentation hardness	EN ISO 2039 [57]	77.6 ± 2	
D shore hardness	DIN 53505 [58]	75 ± 2	
Burning point	DIN 53736 [59]	172–180	°C
Softening temperature	EN ISO 306 [60]	163	°C

**Table 5:** Volume and weight dosages of cement and aggregate in mortars.

C/A Proportion in Volume	Cement (kg)	Aggregate (kg)	C/A Proportion in Weight
1/3	440	1.346	1/3.05
1/4	350	1.422	1/4.06
1/6	250	1.519	1/6.07

**Table 6:** Consistency types and denomination according to EN 1015-3.

Consistency	Denomination	Slump Flow Value (mm)
Dry		<140
Plastic	P	140–200
Fluid	F	>200

**Table 7:** Dosages in weight of the raw materials for each mortar.

Series	Denomination	Aggregates Substituted by Polyamide in Volume (%)	Cement (g)	Aggregate (g)	Polyamide (g)	Water (g)	W/C
Series I	M-3R	0	600	1800	0.0	420.0	0.70
Series I	M-3PA25	25	600	1350	185.2	480.0	0.80
Series I	M-3PA50	50	600	900	370.4	460.0	0.77
Series I	M-3PA75	75	600	450	555.6	523.0	0.87
Series I	M-3PA100	100	600	0	740.8	487.0	0.81
Series II	M-4R	0	600	2400	0.0	433.7	0.89
Series II	M-4PA25	25	600	1800	246.9	533.3	0.89
Series II	M-4PA50	50	600	1200	493.9	546.6	0.91
Series II	M-4PA75	75	600	600	740.8	650.0	1.08
Series II	M-4PA100	100	600	0	987.7	800.0	1.33
Series III	M-6R	0	600	3600	0.0	773.0	1.29
Series III	M-6PA25	25	600	2700	370.4	724.0	1.21
Series III	M-6PA50	50	600	1800	740.8	809.8	1.35
Series III	M-6PA75	75	600	900	1111.2	952.2	1.59
Series III	M-6PA100	100	600	0	1481.2	1110.0	1.85

**Table 8:** Density values of the tested specimens.

Series	Denomination	Density (kg/m^3^)
Series I	M-3R	2080
Series I	M-3PA25	1850
Series I	M-3PA50	1610
Series I	M-3PA75	1350
Series I	M-3PA100	1120
Series II	M-4R	2050
Series II	M-4PA25	1850
Series II	M-4PA50	1540
Series II	M-4PA75	1270
Series II	M-4PA100	950
Series III	M-6R	2020
Series III	M-6PA25	1710
Series III	M-6PA50	1500
Series III	M-6PA75	1080
Series III	M-6PA100	790

**Table 9:** Masonry mortar classes according to the compressive strength.

Class	M1	M2	M5	M10	M15	M20	Md
Compressive strength (MPa)	1	2.5	5	10	15	20	d
d: a compressive strength over 25 MPa, declared by the producer.							

**Table 10:** Rendering and plastering mortar classes according to the compressive strength.

Class	CS I	CS II	CS III	CS IV
Compressive strength at 28 days (MPa)	0.4–2.5	1.5–5.0	3.5–7.5	≥6.0

**Table 11:** Average compressive strength of each mortar denomination.

Series	Denomination	Compressive Strength (MPa)	Standard Deviation
Series I	M-3R	18.30	0.664
Series I	M-3PA25	15.77	0.294
Series I	M-3PA50	14.27	0.084
Series I	M-3PA75	9.54	0.077
Series I	M-3PA100	7.10	0.178
Series II	M-4R	12.22	0.165
Series II	M-4PA25	11.59	0.382
Series II	M-4PA50	8.94	0.267
Series II	M-4PA75	5.28	0.149
Series II	M-4PA100	4.13	0.059
Series III	M-6R	6.32	0.080
Series III	M-6PA25	5.54	0.394
Series III	M-6PA50	3.50	0.702
Series III	M-6PA75	2.95	0.116
Series III	M-6PA100	2.10	0.091

**Table 12:** Cost of the raw materials.

Raw Material	Cost (EUR/kg)
Cement IV	0.12857
Aggregates (Sand)	0.02285
Polyamide	0.00000
Water	0.00042

**Table 13:** Cost for each of the mortar denominations with a zero value for polyamide powder waste.

	Cost (EUR/m^3^ of Mortar)				
Denomination	Cement	Aggregates	Polyamide	Water	Total
M-3R	57.22	30.77	0.00	0.13	88.63
M-3PA25	55.46	22.18	0.00	0.14	77.78
M-3PA50	54.62	14.56	0.00	0.14	69.32
M-3PA75	50.74	6.76	0.00	0.14	57.65
M-3PA100	50.22	0.00	0.00	0.15	50.38
M-4R	45.84	32.59	0.00	0.13	78.57
M-4PA25	44.87	23.93	0.00	0.13	68.93
M-4PA50	44.81	15.93	0.00	0.13	60.87
M-4PA75	41.69	7.41	0.00	0.15	49.24
M-4PA100	35.54	0.00	0.00	0.15	35.69
M-6R	31.96	34.08	0.00	0.13	66.16
M-6PA25	32.30	25.83	0.00	0.13	57.56
M-6PA50	30.46	16.24	0.00	0.13	46.84
M-6PA75	27.28	7.27	0.00	0.14	34.69
M-6PA100	24.65	0.00	0.00	0.15	24.80

**Table 14:** Cost for each of the mortar denominations considering various distance for transporting the polyamide powder waste to the mortar production plant.

Denomination	Cost (EUR/m^3^ of Mortar)				
	in Place of the Waste Material	to 20 km	to 50 km	to 100 km	to 200 km
M-3R	88.63	88.63	88.63	88.63	88.63
M-3PA25	77.78	77.88	78.04	78.31	78.84
M-3PA50	69.32	69.53	69.84	70.37	71.42
M-3PA75	57.65	57.94	58.38	59.11	60.57
M-3PA100	50.38	50.81	51.47	52.56	54.75
M-4R	78.57	78.57	78.57	78.57	78.57
M-4PA25	68.93	69.05	69.22	69.51	70.08
M-4PA50	60.87	61.10	61.45	62.02	63.17
M-4PA75	49.24	49.56	50.04	50.84	52.44
M-4PA100	35.69	36.03	36.60	37.51	39.33
M-6R	66.16	66.16	66.16	66.16	66.16
M-6PA25	57.56	57.68	57.87	58.18	58.80
M-6PA50	46.84	57.07	57.42	48.01	49.18
M-6PA75	34.69	35.00	35.48	36.26	37.83
M-6PA100	24.80	25.18	25.75	26.70	28.59

**Table 15:** Cost for each mortar denominations considering the saving on the waste management.

Denomination	Cost (EUR/m^3^ of Mortar) at 200 km	Additional Profit (Considering 60 EUR/t PA)	Final Cost (EUR/m^3^ of Mortar)
M-3R	88.63	0.00	88.63
M-3PA25	78.84	7.99	70.85
M-3PA50	71.42	15.73	55.68
M-3PA75	60.57	21.93	38.64
M-3PA100	54.75	32.83	21.92
M-4R	78.57	0.00	78.57
M-4PA25	70.08	8.62	61.46
M-4PA50	63.17	17.21	45.96
M-4PA75	52.44	24.02	28.43
M-4PA100	39.33	27.30	12.03
M-6R	66.16	0.00	66.16
M-6PA25	58.80	9.31	49.49
M-6PA50	49.18	17.55	31.63
M-6PA75	37.83	23.51	14.26
M-6PA100	28.59	28.41	0.18

**Table 16:** Summary of the properties (density and compressive strength) and prices of the tested mortar specimens.

Denomination	Density (kg/m^3^)	Compressive Strength (MPa)	Cost (EUR/m^3^ of Mortar) at 0 km ^a^	Cost (EUR/m^3^ of Mortar) at 200 km ^b^	Final Cost (EUR/m^3^ of Mortar) at 200 km Including Profit
M-3R	2080	18.3	88.63	88.63	88.63
M-3PA25	1850	15.77	77.78	78.84	70.85
M-3PA50	1610	14.27	69.32	71.42	55.68
M-3PA75	1350	9.54	57.65	60.57	38.64
M-3PA100	1120	7.10	50.38	54.75	21.92
M-4R	2050	12.22	78.57	78.57	78.57
M-4PA25	1850	11.59	68.93	70.08	61.46
M-4PA50	1540	8.94	60.87	63.17	45.96
M-4PA75	1270	5.28	49.24	52.44	28.43
M-4PA100	950	4.13	35.69	39.33	12.03
M-6R	2020	6.32	66.16	66.16	66.16
M-6PA25	1710	5.54	57.56	58.80	49.49
M-6PA50	1500	3.50	46.84	49.18	31.63
M-6PA75	1080	2.95	34.69	37.83	14.26
M-6PA100	790	2.10	24.80	28.59	0.18

Notes: a: Considering that the polyamide powder waste is generated at 0 km from the mortar production plant; b: Considering that the polyamide powder waste is generated at 200 km from the mortar production plant; c: Considering that the polyamide powder waste is generated at 200 km from the mortar production plant and considering the profit of managing the waste material (60 EUR/t of polyamide).

## Supplementary Materials

The following are available online at https://www.mdpi.com/2073-4360/12/11/2657/s1, Figure S1: (**a**) Dry raw materials, (**b**) Mortar mixing device, Figure S2: (**a**) Compaction device, (**b**) Mould filled with mortar, with 3 specimens, Figure S3: Curing room with various specimens, Figure S4: (**a**) Model MEM-101/SDC of the firm Suxpecar, (**b**) Testing a specimen, Table S1: Compressive strength values of the six specimens tested for each mortar denomination.
