# Supplementary material for: Analysis and Economic Evaluation of the Use of Recycled Polyamide Powder in Masonry Mortars
Source: Polymers (Basel). 2020 Nov 11;12(11):2657. doi: 10.3390/polym12112657 (PMC7697351; doi:10.3390/polym12112657)
Supplement: Supplementary file 1 [file polymers-12-02657-s001.pdf]

Supporting information of:

Analysis and Economic Evaluation of the Use of Recycled Polyamide Powder in Masonry Mortars

Miguel A. Salas [1], Heriberto Pérez-Acebo [2,*], Verónica Calderón [1] and Hernán Gonzalo-Orden [3]

[1] Department of Architectural Construction and Construction and Land Engineering, University of Burgos, 09001 Burgos, Spain; masalas@ubu.es (M.A.S.); vcalderon@ubu.es (V.C.)
[2] Mechanical Engineering Department, University of the Basque Country UPV/EHU, 48013 Bilbao, Spain; heriberto.perez@ehu.eus
[3] Department of Civil Engineering, University of Burgos, 09001 Burgos, Spain; hgonzalo@ubu.es
* Correspondence: heriberto.perez@ehu.eus; Tel.: +34-94-601-7820

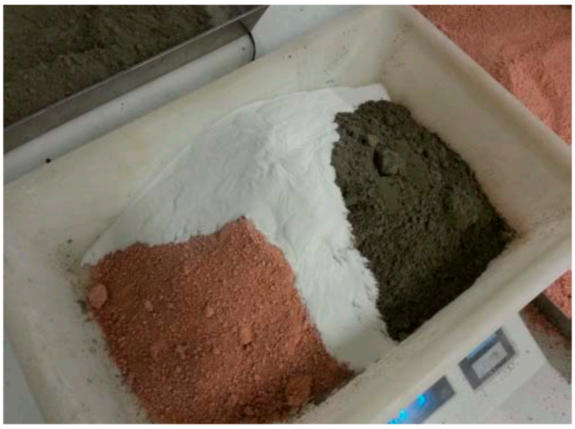

(**a**)

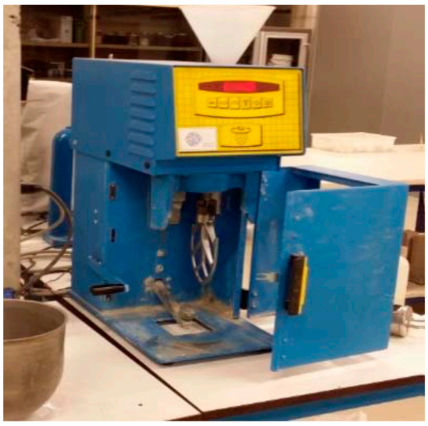

(**b**)

Figure 1. a) Dry raw materials, b) Mortar mixing device.

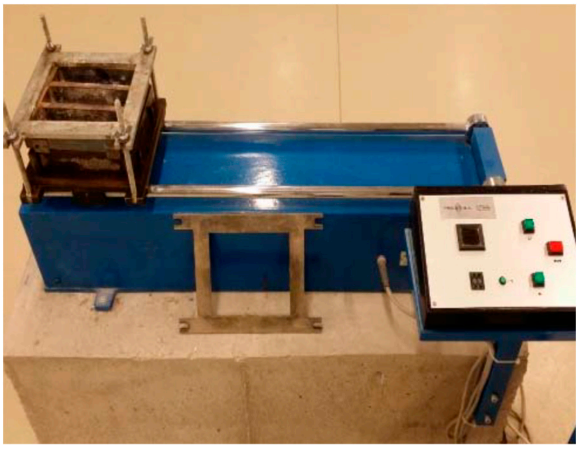

(**a**)

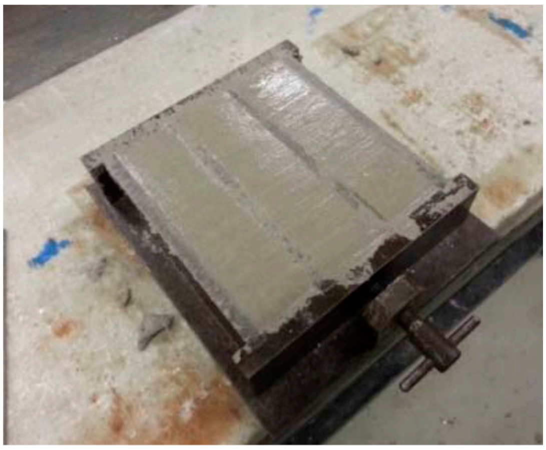

(**b**)

Figure 2. (**a**) Compaction device; (**b**) Mould filler with mortar, with 3 specimens.

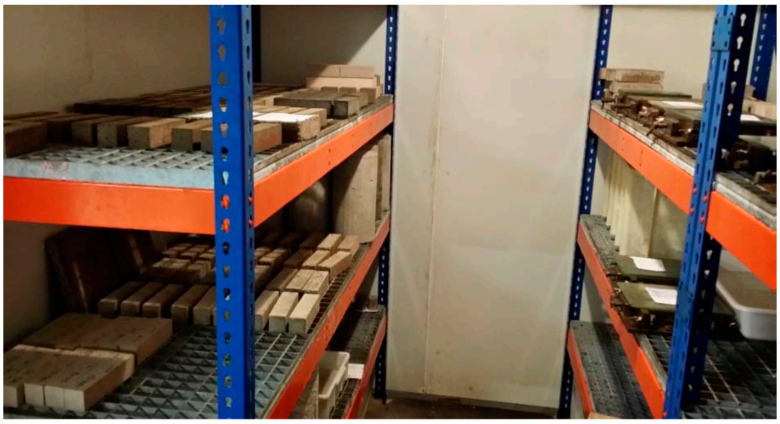

Figure 3. Curing room with various specimens.

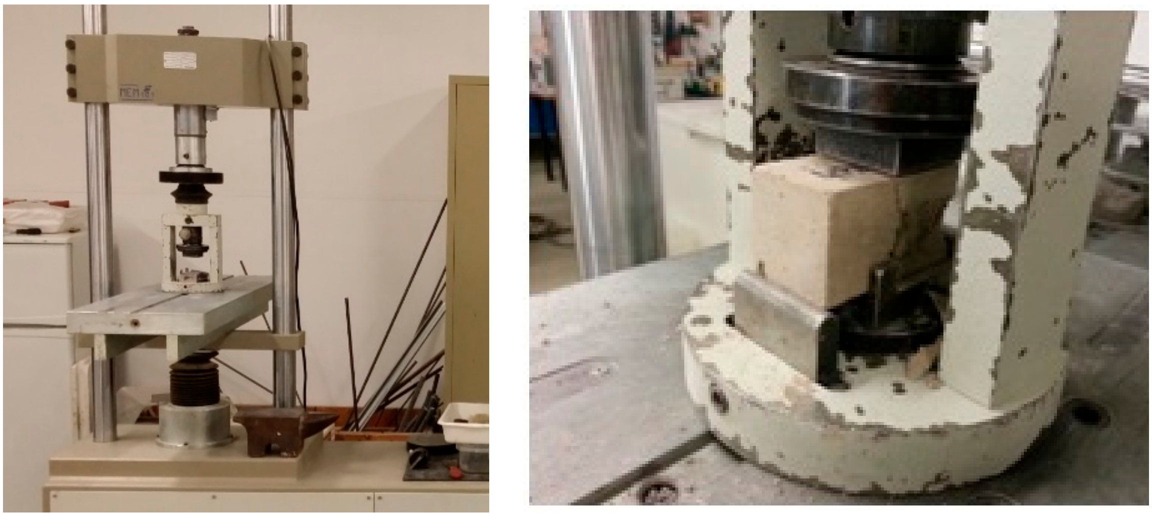

(**a**) (**b**)

Figure 4. (**a**) Model MEM-101/SDC of the firm Suzpecar; (**b**) Testing a specimen.

Table S1. Compressive strength values of the six specimens tested for each mortar denomination.

Denomination	Specimen 1	Specimen 2	Specimen 3	Specimen 4	Specimen 5	Specimen 6	Average compressive strength (MPa)
M-3R	17.70	17.50	18.84	19.36	17.88	18.53	18.30
M-3PA25	15.63	15.77	16.16	16.11	15.66	15.30	15.77
M-3PA50	14.25	14.35	14.13	14.28	14.39	14.23	14.27
M-3PA75	9.63	9.58	9.58	9.41	9.59	9.47	9.54
M-3PA100	7.25	7.15	7.30	7.16	6.78	6.96	7.10
M-4R	12.16	12.08	12.13	12.06	12.47	12.43	12.22
M-4PA25	11.13	11.09	11.69	11.61	12.16	11.87	11.59
M-4PA50	9.06	8.90	8.63	8.57	9.28	9.19	8.94
M-4PA75	5.59	5.32	5.25	5.20	5.17	5.15	5.28
M-4PA100	4.22	4.14	4.16	4.14	4.09	4.03	4.13
M-6R	6.44	6.40	6.34	6.28	6.26	6.21	6.32
M-6PA25	6.11	6.08	5.31	5.24	5.28	5.22	5.54

M-6PA50	3.83	3.79	5.33	3.26	3.41	3.38	3.50
M-6PA75	3.11	3.03	3.02	2.94	2.83	2.78	2.95
M-6PA100	2.00	1.96	2.16	2.14	2.22	2.12	2.10
